# Editorial: Diet and exercise: modulating gut microbiota for enhanced physiological function

**DOI:** 10.3389/fspor.2025.1764433

**Published:** 2026-01-06

**Authors:** Xiaolong Hao, Xin Zhang

**Affiliations:** Department of Food Science and Engineering, Ningbo University, Ningbo, China

**Keywords:** exercise, gut microbiota, health, probiotics, sleep

Modern health science increasingly reveals that regular physical exercise not only directly shapes our physical form and physiological functions, but also profoundly influences multi-dimensional health performances ranging from basic metabolism to advanced neural functions through a complex and delicate internal ecosystem - the gut microbiota.

Sleep quality is fundamental to physical and mental health, yet sleep disorders pose a significant challenge for most people globally. Regular physical exercise, as an effective means of regulating human physiology, has its beneficial effects on sleep well-established. In addition, recent research findings indicate that regulating the gut microbiota is a potential mechanism through which exercise exerts its effects. Advances in microbiome research suggest that the microbiota-gut-brain axis regulates sleep through bidirectional communication between the gut and the brain. Consequently, an interactive network emerges between exercise, gut microbiota, and sleep. Exercise shapes sleep structure by influencing microbial diversity, the production of sleep-related metabolites (such as serotonin, γ-aminobutyric acid, and short-chain fatty acids), and the immune response. Furthermore, factors such as intestinal peristalsis, intestinal barrier function, and bile acid metabolism may affect the gut ecosystem, subsequently influencing sleep. Although the specific mechanism network remains to be fully delineated, employing customized exercise programs to target the regulation of gut microbiota undoubtedly paves a promising non-pharmacological intervention path for addressing the widespread issue of sleep disorders.

The interaction between exercise, diet, and gut microbiota also exerts a profound impact on health outcomes in the elderly population, particularly in terms of glucose and lipid metabolism and chronic inflammation control. Studies have shown that physical activity levels are closely correlated with dietary patterns. For example, moderate-intensity activity is positively associated with the intake of dark green leafy vegetables, while negatively associated with red meat and fried food intake. This “exercise-diet” synergistic pattern jointly affects health biomarkers: high-intensity activity is positively correlated with beneficial high-density lipoprotein cholesterol levels, while moderate-intensity activity is negatively correlated with blood glucose and inflammatory marker C-reactive protein levels. In-depth mediation analysis reveals more detailed mechanistic pathways: the positive effects of moderate-intensity physical activity on reducing C-reactive protein (inflammation) and blood glucose are achieved by increasing the intake of dark green leafy vegetables and vitamin B12, respectively. This finding is crucial, as it indicates that diet is not only a determinant of health alongside exercise, but also an important mediator or “executer” of specific physiological benefits derived from exercise. Exercise may ultimately optimize the regulation of metabolism and inflammation through pathways such as the gut microbiota, by promoting healthier dietary choices or enhancing the body's utilization efficiency of specific nutrients.

The gut microbiota of athletes is not a passive target subject to intervention, but rather a highly dynamic and adaptable active system. Longitudinal monitoring studies on elite volleyball athletes clearly reveal this point. One study has shown that during an eight-week season, although the gut microbiota of athletes remains relatively stable at the phylum level (dominated by *Firmicutes*, *Actinobacteria*, and *Bacteroidetes*), its internal composition adjusts sensitively with training load and competition rhythm. Specifically, the ratio of *Firmicutes* to *Bacteroidetes*, which reflects energy intake and storage potential, increases during regular training and competition periods and decreases during rest periods. It is particularly noteworthy that the abundance of *Ruminocoreaceae*, which is associated with recovery and anti-inflammatory processes, significantly increases during recovery. These regular fluctuations suggest that specific microbial population changes, such as the *Firmicutes*/*Bacteroidetes* ratio and the abundance of *Ruminocoreaceae*, may become novel microbial biomarkers for objectively assessing training physiological load and monitoring recovery status. This discovery elevates the gut microbiota from the role of a health “indicator” to a potential “participant” and “monitor” in sports physiological regulation.

Probiotics, as a mechanism for enhancing athletic performance, have been further elucidated at the micro level in animal model studies. Taking *Lactobacillus brevis* GKEX as an example, research has shown that supplementation with this strain, whether in live or inactivated form, significantly improves the exercise endurance of mice, manifested as enhanced forelimb grip strength and prolonged exhaustive running time. The biochemical basis of its anti-fatigue effect lies in its ability to effectively promote the clearance of post-exercise lactic acid, increase glycogen reserves in the liver and muscles, and simultaneously reduce blood lactate production and blood urea nitrogen levels - all of which are key indicators for measuring fatigue levels and recovery speed. More significantly, the exercise benefit is accompanied by the remodeling of gut microbial ecology, particularly the promotion of the proliferation of short-chain fatty acid-producing bacteria. This strongly suggests that probiotics may systematically improve energy metabolism and recovery processes by regulating the composition of gut microbiota and its metabolic output (such as increasing short-chain fatty acids), thereby enhancing overall athletic performance and anti-fatigue capabilities. It provides solid experimental evidence for the development of exercise nutritional supplements based on specific strains.

The association between the microbiota and physical activity has become a key frontier in sports performance research, with the impact of probiotics on athletes being a relatively new area of investigation. Studies have found that probiotics appear to benefit adults in terms of mental health, cognitive function, sleep, gastrointestinal health, and upper respiratory symptoms. Furthermore, when designing training programs, the supplementation of probiotics and its impact on different types of athletic performance are crucial. In most cases, probiotic supplementation is effective in both major types of sports: probiotics have strain- and duration-specific effects on both endurance and interval-related sports. Supplementation with probiotics can reduce inflammatory process activity and stress-related factors, such as anxiety and depression, in interval-related sports. In endurance sports, probiotics can enhance lipid metabolites, including short-chain fatty acids and polyunsaturated fatty acids, regulate maximum oxygen uptake, and reduce gastrointestinal symptoms. Exploring the relationship between probiotics and athletic performance can provide valuable insights for professional athletes to optimize training techniques and strategies.

In summary, moderate physical exercise positively impacts gut biodiversity, boosting the population of beneficial bacteria crucial for energy metabolism, immune responses, and neuroendocrine regulation. Despite these advances, the intricacies of how gut health influences overall physiological outcomes, particularly in response to exercise, remain a field of active investigation with considerable gaps in comprehensive mechanistic understanding. Future research requires deeper interdisciplinary collaboration to elucidate the precise mapping relationships between different exercise modes, intensities, durations, and specific bacterial strains, metabolic pathways, thereby promoting the development of truly personalized, microbiota-based sports medicine and health promotion strategies.

